# The Role of Residential Treatment in Addiction Care

**DOI:** 10.1177/14550725261479851

**Published:** 2026-08-30

**Authors:** Joar Guterstam

**Affiliations:** 1 Centre for Psychiatry Research, Department of Clinical Neuroscience, Karolinska Institutet & Stockholm Health Care Services, Region Stockholm, Stockholm, Sweden

One of the most common misconceptions about addiction care is that it typically involves residential treatment, or “checking into rehab.” In reality, most countries primarily offer outpatient services to patients seeking help for substance use disorders, and residential treatment is reserved for selected cases, where other options have failed. This stepped-care model may seem rational, considering the costs associated with residential treatment. However, the limited evidence regarding the pros and cons of such options makes it hard to decide when and for whom residential treatment would be beneficial (de Andrade et al., 2019). Although intense treatment in a drug-free environment can be a life-saving intervention for one person, the disruption of everyday contacts with friends, family, workplace, and long-term caregivers that residential treatment often entails, can actually make the situation worse for another person.

This knowledge gap about one of the most expensive treatments available in addiction care is problematic. The study by Høiland et al. (2026) describes current practice in Norwegian addiction care, by characterizing the patient populations referred to outpatient and residential treatment, respectively. The authors found that higher severity of alcohol use disorder was associated with referral to residential treatment, whereas other clinically relevant factors such as psychiatric and somatic co-morbidity did not differ significantly from the patient group referred to outpatient treatment.

These findings should be interpreted with caution, considering the limited sample size and lack of information on earlier treatment history, but may nevertheless inspire further work and discussion about the role of residential treatment in modern addiction care. Ideally, one would wish for large randomized clinical trials on this topic, but other forms of evidence can also be informative, especially cohort studies that may capture relevant long-term outcomes. The population-based registers of the Nordic countries provide great opportunities for this, which can hopefully be explored more in future work.

A special and highly controversial case is involuntary residential treatment for addictive disorders. This is still used in Sweden, where patients can be kept in coercive care for up to 6 months under the Care of Substance Abusers (Special Provisions) Act (LVM). The pros and cons of such measures have been debated, and recent Official Reports from the Swedish government have suggested abolishing this system, allowing only for brief, hospital-based involuntary treatment under the law governing coercive psychiatric care (Från delar till helhet, 2023; Bättre insatser vid missbruk och beroende, 2011). These suggestions are primarily based on ethical concerns with the current practice, but it is unfortunate that the scientific evidence guiding the decisions is so weak and inconsistent. The lack of relevant studies of the current system will also make it hard to assess the effects of new legislation in the future. From this perspective, even relatively simple observational studies can be of value.

Because residential treatment, whether voluntary or not, is costly and aimed at some of our most vulnerable patients, further research on this topic should be given high priority.
